# Possibility to Grasp the Older Drivers’ Conditions from the Triennial Nationwide Survey of Japan for Elderly Welfare

**DOI:** 10.3390/ijerph22010005

**Published:** 2024-12-24

**Authors:** Mengmeng He, Yasuhiro Yamanaka, Kazuya Takamatsu

**Affiliations:** 1 Graduate School of Environmental Science, Hokkaido University, Sapporo 060-0810, Japan; 2 Faculty of Environmental Earth Science, Hokkaido University, Sapporo 060-0810, Japan; galapen@ees.hokudai.ac.jp; 3 Tsurui Village Office, Tsurui 085-1203, Japan; kazuya_takamatsu@vill.tsurui.lg.jp

**Keywords:** older adults, driving cessation, *kihon* checklist (KCL), must-watch drivers, needs survey

## Abstract

The percentage of older drivers is increasing worldwide. Older adults are driving for their daily lives, including drivers who should not drive, “must-watch drivers”, for health conditions, etc. The “Public Survey of Long-Term Care Prevention and Needs in Spheres of Daily Life (Needs Survey)”, including the “*Kihon* Checklist (KCL)”, is a triennial nationwide survey conducted by welfare administrations in Japan. The objective of this study was to demonstrate that the Needs Survey can capture situations (e.g., driving avoidance) of older drivers obtained by previous studies, many of which are one-time surveys. As for our methods, we administered a survey with a format of questions used in previous studies combined with KCL to all older adults in Tsurui Village, a rural community in Japan, obtained 393 responses, around half of them, and conducted a logistic regression analysis to estimate whether they were driving or not and a multiple regression analysis for the frequency of driving avoidance. The former analysis showed that KCL could detect must-watch drivers with relatively deteriorated health among not-so-old adults, adding to another one with relatively not-so-bad health among much older adults, and the latter analysis showed that the KCL scores could be an alternative to the self-rating of driving ability used in previous studies. In conclusion, KCL in the Needs Survey is recommended to be a valuable survey for regularly assessing the driving conditions of older drivers nationwide.

## 1. Introduction

The older adult population ratio, the percentage of adults aged 65 and older in the total population, is expected to increase from 10% in 2022 to 16% in 2050 worldwide [1]. In developed countries, many older adults drive cars alone. The driving rates of older adults in Japan, USA, and Germany are 54.9%, 87.0%, and 67.7%, respectively [2]. With aging, driving ability declines due to age-related factors. For example, they may be unaware of illnesses that negatively affect driving [3]; older drivers with visual acuity and hearing deficits are at a greater motor vehicle collision (MVC) risk [4]; high dosages of prescription and nonprescription drugs can make safe driving hazardous [5]; they may suffer from cognitive impairment (executive functioning, global functioning, visuospatial skills, reaction times) [6,7,8]. With driving cessation, older adults may become lonely due to the loss of social networks [9,10] or suffer damage to their health, such as depression [11]. In the process of driving cessation, older drivers become more likely to avoid driving [12] and to avoid challenging driving situations, such as in rain, during nights, at dawn or dusk, during nights while it is raining, first-time visits, and on highways due to self-regulation, lifestyle changes, or preference [13,14,15,16,17]. Even if older drivers avoid driving, they cannot reduce their risk of encountering traffic accidents [18], and cautious driving skills are insufficient to compensate for older drivers’ functional decline [19]. Few older adults are prepared for driving cessation, even if it is likely to happen [20]. Safer alternative transport options must be provided to older adults wherever possible, considering the extent of their daily lives [21]. Older adults do not like to use public transportation from the perspective of affordability, accessibility, and safety [22]. For older adults who cease driving, we need to provide an age-friendly, sustainable transportation system that takes into account these points [22]. Therefore, comprehensive and early identification of older drivers, whose ability to drive is becoming more difficult, is crucial to support older drivers.

The older population ratio in Japan was 29.0% in 2021 and will be 35% in 2040, the most aged population among developed countries [23]. In total, 54.7% of older adults have a driver’s license [24], and in rural areas, about 80% of older adults are dependent on cars [25,26,27]. In Japan, the “*Kihon* checklist (KCL)” is often used as a screening tool to identify older adults who need support in long-term care insurance certification [28,29]. KCL was also used in the “Public Survey of Long-Term Care Prevention and Needs in Spheres of Daily Life” (hereinafter referred to as the “Needs Survey”), the standard format in all 1718 municipalities in Japan conducted as municipalities were developing “Municipal Insured Long-Term Care Service Plan” [30]. Moreover, small municipalities survey all older adults.

KCL has been used in academic fields related to welfare, but only two studies have applied KCL to older drivers: some current drivers should not drive based on their stage, gender, and health conditions, named as “must-watch drivers” [31], and older adults who are pre-frail are 1.52 times (95% confidence interval 1.10–2.10) more likely to be involved in a traffic accident than healthy older drivers [32]. Since these studies [31,32] did not include the questionnaire items (e.g., self-rating of driving ability) that many previous studies (e.g., [15,33,34]) used, they could not present whether the KCL would improve older drivers’ driving avoidance.

The classification model suggested that the contextual-mediated model, in which *distal* context (i.e., demographics, personality, etc.) influences the likelihood of being involved in MVCs both directly and indirectly through the mediation of and via *proximal* context (i.e., driving behaviors and driving skills) [35], is applicable in the analysis of determinants of MVCs in different populations of drivers [36].

**Figure 1:**
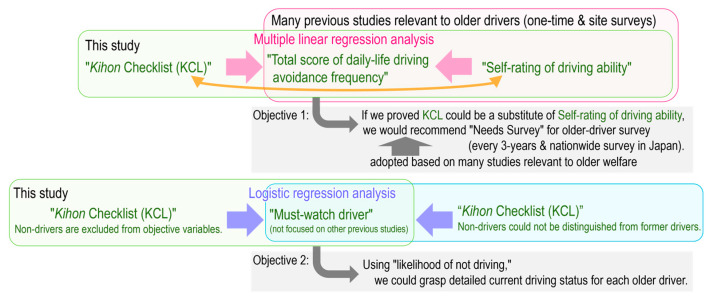
Schematic diagram of the analysis and purpose of this study and their relationship to previous studies.

The objective of this study was to demonstrate that the Needs Survey, especially KCL scores as a proximal factor to substitute the self-rating of driving ability, can capture situations (e.g., driving avoidance) of older drivers obtained by previous studies (e.g., [12,13,14,15,16,17]), many of which are one-time surveys, as a simulated survey of the Needs Survey nationwide and every three years conducted by welfare administrations (Figure 1). If this study can demonstrate this, the Needs Survey will enable the nationwide implementation of policies, such as screening of must-watch drivers [31] and safer alternatives [20], without conducting additional surveys on the older drivers’ driving status, driving or not driving, in each municipality. Another objective of this study was to investigate how to better understand the must-watch driver, a classification used by the Needs Survey, presented by He et al. [31] (Figure 1).

## 2. Materials and Methods

In Tsurui Village, a rural area in Hokkaido, Japan (population, 2489; area, 571.8 km^2^; the proportion of older adults, 33.3% in September 2022), we conducted a survey asking questions about driving avoidance and KCL to all residents aged 65 and older, 829 people, on 1 October 2022, in collaboration with the Tsurui Village office. Our research was approved by the Ethics Review Board of the Faculty of Environmental Earth Science, Hokkaido University (approval date: 28 September 2022). We posted 829 self-administered questionnaires to all residents aged 65 and older and received 393 valid responses (response rate of 47.4%) between 19 October and 15 November 2022. The collected data were analyzed using methods comparable to those in previous studies [15,31,33,37,38].

### 2.1. Survey Items and Questionnaire Content

The questionnaire consisted of the following questions based on previous studies and the Needs Survey. As used in previous studies [15,39], the questions were designed to assess the driving avoidance frequency across the seven challenging driving situations: (1) rain, (2) nights, (3) dawn or dusk, (4) nights during rain, (5) long time (more than 2 h one way), (6) first time in place, (7) highways. For each condition, we asked, “In the past year, how often have you avoided driving?” The respondents were asked to choose one of five options: never, rarely, sometimes, often, and always. Referring to previous studies [33,40], we asked about their evaluation of the importance of driving in their daily lives and self-rating of driving ability by choosing one of five options: from “very important” to “not important at all” and from “excellent” to “poor”, respectively.

Respondents were also asked about their current driving frequency with the following question, “How often do you currently drive yourself?”, by choosing one of six options as follows: nearly daily, 3–5 times weekly, 1–2 times weekly, 2–3 times monthly, not driven in the past six months, and no driver’s license/no car, referring to previous studies [34,41]. We added the same question using “in your 50s” instead of the word “currently” without the option “not driven in the past six months”. We classified respondents choosing the last option, no driver’s license/no car, both for the current (in many cases, those who have never driven in their lives) and their 50s as “non-drivers” and respondents choosing the last option only for the current as “former drivers” after stopping driving in their 50s, which were not be distinguished in many previous studies (e.g., [31]). We also asked about the transportation options they use when going out. The answers were as follows: (1) driving themselves, (2) riding in a car driven by others, and (3) using public transportation such as buses.

KCL consists of 25 questions defined by Arai et al. [42], listed in Appendix A, to assess the following domains as categorized by Fukutomi et al. [43]: the instrumental activities of daily living (IADL) (questions 1–5), physical (questions 6–10), nutrition (questions 11–12), oral (questions 13–15), socialization (questions 16–17), memory (questions 18–20), and mood (questions 21–25). Respondents answer “yes” or “no” (with “yes” indicating good health for most questions, but “no” indicating good health for some questions). We calculate the total number of no-good health options using the KCL scores, and we judge older adults with higher KCL scores as having issues with their functional living capabilities. Satake et al. [44] revealed the relationship between KCL scores and frailty measures: 0 to 3 for robust individuals, 4 to 7 for prefrailty, and 8 or higher for frailty. Frailty measures are widely used worldwide (e.g., [45]), although they are linked to frailty measures by Satake et al. [44] in Japan.

We also asked for their demographic information: gender, age (in 5-year increments), bus route area, and family structure (living alone, living with a spouse, or living with family such as their son/daughter). Since many respondents could be identified by their residential area, age per year, and gender, we asked about age in increments of 5 years for the privacy of respondents.

### 2.2. Data Analysis

We used two main analyses and one supplemental analysis. Statistical significance was defined as *p* < 0.05 using R version 4.2.0 with the ggplot2, dplyr, and stargazer packages [46].

The first main analysis is the logistic regression analysis to guess the driving status, driving/not driving, with former and current drivers as objective variables and explanatory variables: gender, bus route area, and living alone as categorical variables, and age and KCL scores as quantitative variables (Model 1). As frailty measures (robust, pre-frailty, and frailty based on KCL scores) are used in the academic welfare field, following Liu et al. [32], we calculated the frailty measure as a categorical variable instead of the KCL scores as an explanatory variable (Model 2). In addition, we also calculated using neither the KCL scores nor the frailty measures (Model 3) and using only age, gender, and KCL scores as explanatory variables, respectively. By comparing Model 1 (including KCL scores as an explanatory variable) with Model 3 (not including KCL), we show how much the predictability of KCL, a substitute for the self-rating of driving ability as a *proximal* variable, improves compared to the case of only *distal* variables. We also show that we can discuss identifying must-watch drivers using Model 2 (using frailty measures, widely used in the welfare administrations, as explanatory variables).

As for the supplemental analysis, Spearman’s rank correlations of the driving avoidance frequencies as ordinal data for each situation were obtained for the seven challenging driving situations used in previous studies. Based on this, we selected four “challenging daily-life driving situations” (in rain, during nights, at dawn or dusk, during nights while it is raining) and calculated the “total score of daily-life driving avoidance frequency.”

To identify the factors influencing driving avoidance of current drivers focused on previous studies (e.g., [15,34,37]), the second main analysis was the multiple linear regression analysis between the “total score of daily-life driving avoidance frequency” and multi-variables: gender and living alone as categorical variables and current driving frequency, self-rating of driving ability, and evaluating the importance of driving as ordinal variables scored into quantitative variables; and age and KCL scores as quantitative variables. The “total score of daily-life driving avoidance frequency” is the total score (from 4 to 20 points) of the ordinal data of the five choices for four “challenging daily-life driving situations” with the scores as follows: 1 point for never, 2 points for rarely, 3 points for sometimes, 4 points for often, and 5 points for always. Since we focused on the self-rating of driving ability used in previous studies (e.g., [15,33,34]) and KCL scores included in the Needs Survey, Model A used both variables; Model B used only the former; Model C used only the latter, and Model D used no variables, with other variables: gender, age, and living alone. The explanatory variables for Models A, B, C, and D are adopted to be those that are statistically significant in the multiple regression analysis from Model E in Appendix A with all information as explanatory variables.

In our two main analyses, we compared several models with different numbers of explanatory variables. To determine which was better, we used the Akaike information criterion (AIC). The AIC is used to “balance the complexity of the model and the goodness of fit to the data”, and the model with the lowest AIC value is judged to be the best.

## 3. Results

### 3.1. Basic Statistics of Respondents

**Figure 2:**
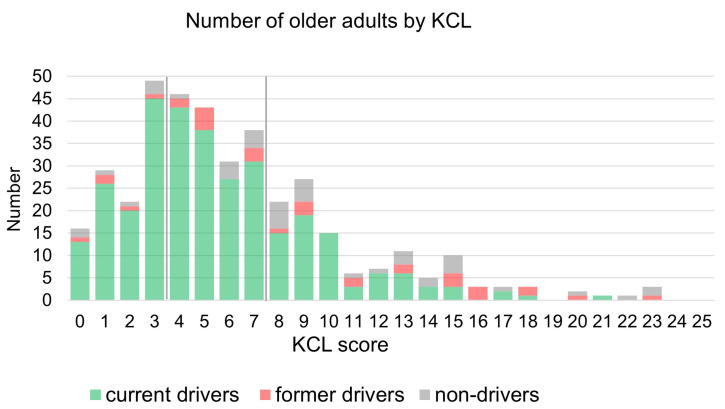
The number of current drivers, former drivers, and non-drivers to KCL scores.

The 393 respondents consisted of 317 current drivers (80.7%), 33 former drivers (8.4%), and 43 non-drivers (10.9%) (Table 1). The number of current drivers decreases with the age increase, with a more significant proportion of the respondents being younger than former drivers and non-drivers. The frailty rates of the total respondents were 23.3%, 54.5%, and 62.8% for current drivers, former drivers, and non-drivers, respectively. The mean (standard deviation) of the KCL scores were 5.44 (3.60), 9.58 (6.12), and 9.67 (5.87) for current drivers, former drivers, and non-drivers, respectively. Current drivers had significantly lower scores than former drivers and non-drivers (Kruskal-Walli’s test, *p* < 0.01) (Figure 2).

### 3.2. Logistic Regression Analysis on Current and Former Drivers With/Without KCL Data

To present how health conditions derived from the KCL data influence the predicted driving status, we compared three logistic models with explanatory variables treated differently for KCL (Table 2). Models 1, 2, and 3 have relatively similar odds ratios and AIC, though these are better than when calculated independently: 163.24 only for age, 219.17 only for gender, and 197.21 for KCL scores. In Model 1, the likelihood of not driving is six times (OR = 6.011, 95% CI [1.989–21.763]) higher for females than males, three times as age increases by five years (OR = 3.056, 95% CI [2.612–6.776]), and one time (OR = 1.160, 95% CI [1.059–1.277]) higher if the total score of KCL is one point higher.

**Figure 3:**
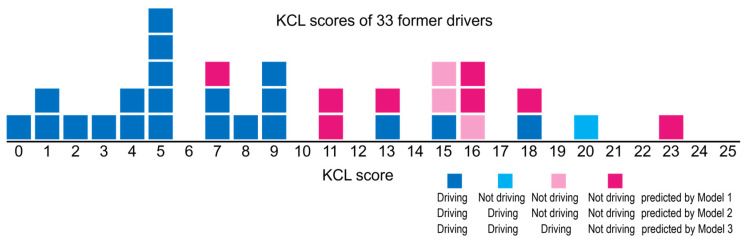
The KCL scores and predictions on driving by Models 1, 2, and 3 for 33 former drivers.

The “correct response rate” where the predicted driving/not driving matches the current/former drivers is the number of diagonal components for all respondents, 92.3%, 92.0%, and 92.0% for Models 1, 2, and 3, respectively. The number of must-watch drivers, current drivers but not driving, predicted by models are 6, 6, and 3 people by Models 1, 2, and 3, respectively (Table 3). Also, former drivers but driving predicted by Models 1, 2, and 3 are 21, 22, and 25, respectively. These are former drivers with relatively small KCL scores among former drivers (Figure 3). The difference in the number of former drivers but driving predicted by Models 1, 2, and 3 is due to different projections for former drivers with high KCL scores.

Table 3 shows the number of drivers divided by 0.5 as a threshold to judge whether they are driving or not driving, and the numbers of must-watch drivers for Models 1, 2, and 3 do not change significantly, as shown in Table 3. However, the likelihood of older drivers around the threshold of 0.5 dramatically changes depending on the KCL, and therefore, we can find older drivers who are closer to must-watch drivers. Comparing Models 1 and 3, we can also find must-watch drivers due to their high KCL.

**Figure 4:**
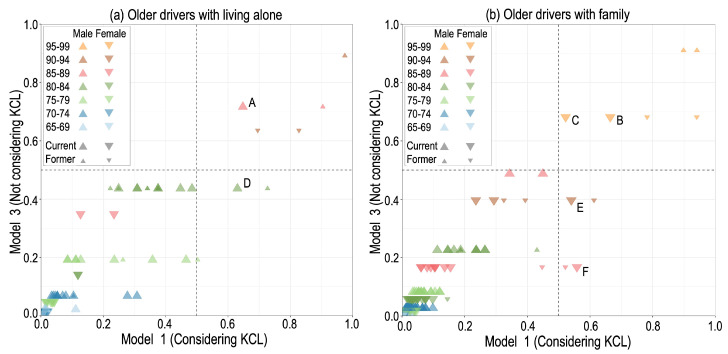
The likelihood of not driving by considering KCL (Model 1)/not considering KCL (Model 3), (**a**) for older drivers living alone and (**b**) for older drivers with family. The older drivers with A–F are quoted in the main text.

The likelihood of not driving using KCL scores (Model 1) differs from that without considering any health conditions (Model 3) (Figure 4). Whether considering KCL or not, the likelihood of not driving increases with age for both males and females. The likelihood of not driving for females aged 80–84 in Model 3 includes the following: 0.44 for respondents living alone (Figure 4a) and 0.24 for respondents living with others (Figure 4b). There is a horizontal distribution due to considering KCL in Figure 4, even for the same gender and age group. Interestingly, the distributions around 0.5, the threshold value judging whether they do not drive, are outstandingly large spread values. For example, two groups with a likelihood of not driving are around 0.44 and 0.40 without KCL considered (Model 3), i.e., 80–84-year-old females living alone (green ▲ in Figure 4a) and 90–94-year-old males living with others at 0.40 (brown ▼ in Figure 4b), expand in a range of approximately 0.4 ± 0.2 with KCL considered (Model 1).

As a result, two older drivers (Ms. D and Mr. E) were judged not to drive, with the likelihood exceeding 0.5 in Model 1, due to their high KCL scores of 12 and 13, respectively. Similarly, in a group with a low likelihood of not driving based on their age, gender, and status of living alone in Model 3, one older driver having a high KCL score of 21 exceeded the likelihood of 0.5 (Mr. F), who is identified as a must-watch driver by regression analysis using KCL only. Additionally, focusing on the two groups in Model 3 with the likelihood of not driving around being 0.7, two males (Mr. B and Mr. C) aged 95–99 live with others with a probability of 0.68 and a single female (Ms. A) aged 85–89 with a probability of 0.71 are must-watch drivers with KCL scores of 5, 9, and 5, respectively, which means relatively not-so-bad health conditions but their likelihood of not driving never being below 0.5.

### 3.3. Driving Conditions and Avoidance of Current Drivers

#### 3.3.1. Driving Conditions of Current Drivers

About 90% of females and males drive at least once a week and consider it very important or important for their daily life (Table 4). Females have a lower driving frequency, lower importance of driving, and higher riding in a car driven by others. No gender differences are observed in the self-rating of driving ability.

**Figure 5:**
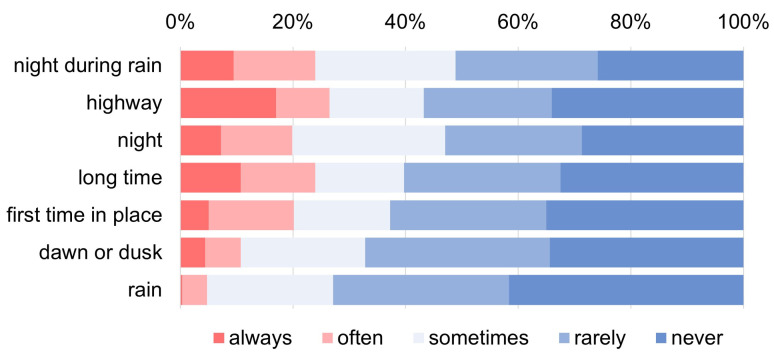
The driving avoidance frequencies in seven challenging driving situations are sorted in order of difficulty based on responses. Highways tend not to be used in remote areas for more than two hours one way.

**Figure 6:**
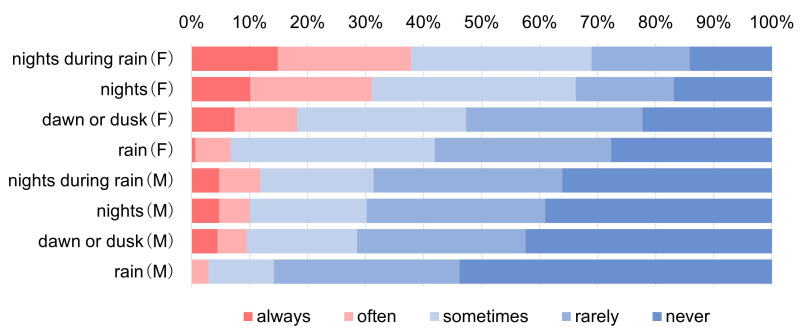
Driving avoidance frequency in four “challenging daily-life driving situations” by gender (for situations, responses are scored and sorted in order of difficulty).

**Figure 7:**
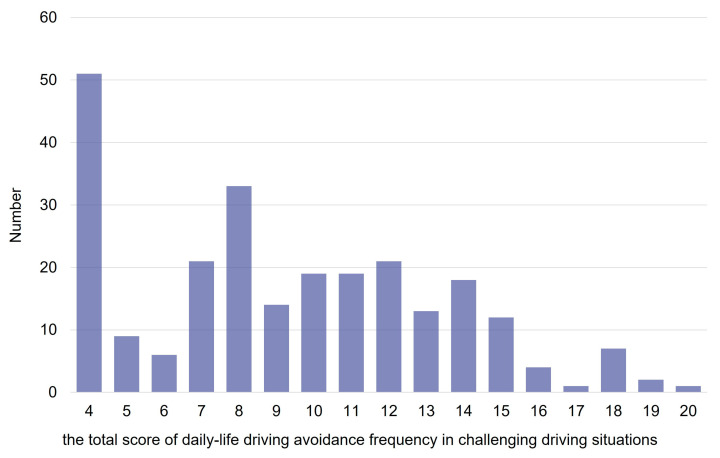
Number of current drivers by the “total score of daily-life driving avoidance frequency” in challenging driving situations.

While most current drivers drive during the day regardless of weather conditions (5% of current drivers often avoid driving in the rain), 20% (24%) of current drivers do not drive at night (nights during rain) (Figure 5). When calculating Spearman’s rank correlations of driving avoidance frequency among seven challenging driving situations (Table 5), the correlations among nights, nights during rainy, and dawn or dusk are high (over 0.79), and the correlations in the rain with these three challenging driving situations are the next highest (0.71 to 0.63). As for the three challenging non-daily-life driving situations, such as on highways, for a long time (more than 2 h one way), and first time to a place, the correlations among them are also high (0.72 to 0.63), with about 20% of current drivers taking driving avoidance at night. Any one of three challenging non-daily-life driving situations has weaker correlations with four “challenging daily-life driving situations” (0.64–0.49), possibly due to factors different from daily life. This study will focus on four “challenging daily-life driving situations”: rain, night, dawn or dusk, and night during rain. More than 30% of females avoid driving at night (38% during rain), while only 10% of males avoid driving at night (11% at night during rain) (Figure 6). Gender differences are observed at night, at night during rain, and at dawn or dusk. The “total scores of daily-life driving avoidance frequency” for 317 current drivers are widely dispersed, which could be dealt with as pseudo-quantitative data, although the number of drivers not avoiding at all is the largest, 51 (16.1%) (Figure 7).

#### 3.3.2. Multiple Regression Analysis for Driving Avoidance Frequencies of Four “Challenging Daily-Life Driving Situations” Based on the Health Conditions of the Older Drivers

To clarify the driving avoidance frequencies of the four “challenging daily-life driving situations”, we conducted multiple regression analyses between driving avoidance frequencies and other variables with four models: Model A used both the self-rating of driving ability and KCL scores; Model B used only the former; Model C used only the latter, and Model D used no variables, with other variables: gender, age, and living alone (Table 6).

The adjusted correlation coefficient and AIC values decrease and increase with Models A, B, C, and D: 0.279 and 1697 for Model A, 0.242 and 1712 for Model B, 9.237 and 1714 for Model C, and 0.165 and 1742 for Model D. These similar results using either one as an explanatory variable (Models B and C) suggest that the self-rating of driving ability or KCL scores may substitute for each other. However, better results in Model A compared to other models also suggested that the self-rating of driving ability or KCL scores does not necessarily represent the older adult’s actual health conditions due to their perceptions of their health conditions.

In situations such as at night, during nights while it is raining, and at dawn or dusk in Model A, the likelihood of driving avoidance is 2.926 times (95% CI [2.139–3.712]) for females compared to males; 0.425 times (95% CI [0.119–0.731]) greater for age increases by five years; 1.1 times (95% CI [0.129–2.071]) higher for those living alone than for other family structures; and 0.236 times (95% CI [0.124–0.348]) higher by increasing one point on KCL scores. The likelihood of driving avoidance by their self-rated driving ability is 1.161 times (95% CI [0.642–1.681]). These results for gender, age, and self-rated driving ability are consistent with the trends in previous studies [15,33,37,38].

## 4. Discussion

We discussed the relationships between the likelihood and the driving status of must-watch drivers and between KCL and self-rating of driving ability, along with two objectives. Through those discussions, we suggested that information on individual likelihood is informative and that KCL can substitute for self-rating of driving ability, which many studies have used.

Focusing on the proportion of frailty in current and former drivers shows that one-fourth of current drivers have declining health, while just under 40% of former drivers are not in deteriorating health (Table 1). In other words, the picture of “driving = good health” is only a loose relationship.

We found six must-watch drivers in Model 1 (Table 1), which is a smaller number than those in He et al. [31], 17–24 must-watch drivers may be different as our survey was 5 years after the survey by He et al. [31]. This is because non-drivers were also included in He et al. [31], while non-drivers were excluded from the objective variable in this study. Using Model 1, there were 17 must-watch drivers when non-drivers were included in the objective variable. The added 11 must-watch drivers were all female. Since 93% of the non-drivers were female (Table 1), and their likelihood was lower than that of former drivers, the OR for gender was higher at 9.988 (95% CI [4.359–25.695]), and their likelihood exceeded the threshold of 0.5.

This study focused on the likelihood of not driving, which provides more detailed information on the driving status of individual older drivers (Figure 4) rather than must-watch drivers judged separately by the threshold of 0.5 (Table 3). We can use likelihood to identify older drivers who are slightly below the threshold of 0.5, the preliminary group that will become must-watch drivers if the conditions change slightly and to judge the severity of must-watch drivers by the degree to which it exceeds the threshold of 0.5. In general, the likelihood of not driving increases with age, and this likelihood for females is comparable to those of males ten years older. When considering KCL, the likelihood of not driving has scattered value due to health conditions (Figure 4), even if age and gender are the same.

Generally, must-watch drivers are categorized into two types: current drivers in relatively deteriorated health among the not-so-old group and relatively not-so-bad health among the much older group. The latter group can be detected based on age and gender without KCL, while the former can only be detected using KCL. Furthermore, the likelihood of must-watch drivers is very scattered, ranging from 0.17 to 0.72 in Model 3, but around 0.6, ranging from 0.52 to 0.66 in Model 1 (Figure 4). This means that with Model 1, the likelihood could be more correctly evaluated for must-watch drivers.

The must-watch drivers have higher KCL scores and living alone statuses, which are closer to the former drivers, although the bus route area, self-rating of driving ability, evaluation of the importance of driving for their daily lives, “total score of daily-life driving avoidance frequency”, and the usual driving destination are not statistically different from those of other current drives (Table 7). Former drivers are more likely to ride in a car driven by others than in public transportation. They may avoid using public transportation from the perspective of affordability, accessibility, and safety [22]. In the items on the percentage of drivers who use public transportation and ride in a car driven by others, the must-watch drivers are closer to other current drivers in the present but closer to former drivers in the future (Table 7). In other words, the must-watch drivers may realize that they must stop driving in the near future. This result would be missed in the simple summation of the questionnaire because of the overwhelming number of other current drivers.

Since it is a crucial decision for older adults, given the health deterioration associated with driving cessation and the risk of traffic accidents caused by continued driving [10,18], the decision should not be based solely on KCL based on the respondents’ self-perceptions. Individualized solutions to the six must-watch drivers would be necessary, including confirmation of their actual driving conditions and securing alternative transportation such as public bus, shuttles to and from the clinic, and volunteer-driving cars. These age-friendly, sustainable transportation systems provided by the administration will be taken into account for affordability, accessibility, and safety [22].

**Figure 8:**
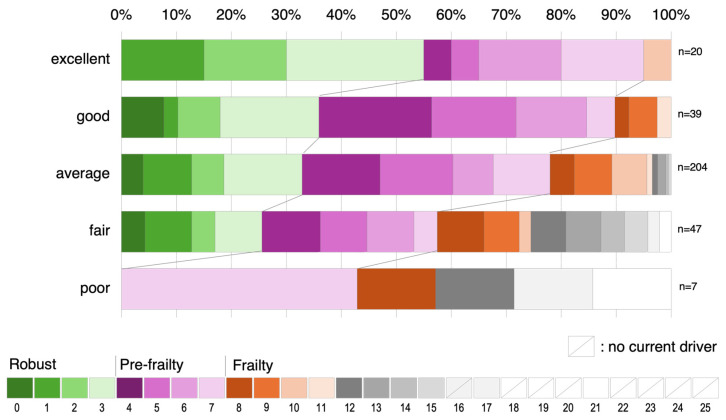
Percentage of KCL scores by the self-rating of driving ability showing the relationship between the KCL scores and self-rating of driving ability.

As shown in Section 3.3.2, adding the information from the self-rating of driving ability and KCL results in a better correlation of the multiple regression analysis with “total score of daily-life driving avoidance frequency”. Since many older adults answered their self-rating of driving ability to be average and their KCL scores ranged widely from 0 to 11 (Figure 8), the relationship between self-rating of driving ability and KCL has not a strong but weak correlation coefficient (Pearson’s correlation coefficient 0.29). As a result, even when using KCL (Model C) used in the Needs Survey as an alternative to the self-rating of driving ability (Model B) used in previous studies, the correlation coefficient for driving avoidance is as high as that for the self-rating of driving ability. Even if current drivers have the same self-rating of driving ability, they are in very different health conditions, at least based on KCL scores. Because KCL has different characteristics from the self-rating of driving ability, it cannot be ruled out that their KCL scores could be higher due to questions that do not affect driving. However, it also suggests that current drivers may overestimate their driving ability more than their actual rating (despite the poor health conditions indicated by the KCL). In this point, there is an advantage in using the KCL scores, which asks about various daily living activities and is a detailed quantification.

This study involved several limitations. The KCL is widely utilized in the welfare field and has been adopted in the Needs Survey conducted nationwide. However, because the KCL scores are based on respondents’ self-perceptions, there is a possibility of discrepancies between their self-perception and the actual diagnosis by professionals. In particular, respondents may not be able to accept a situation in which they must cease driving and may respond to the situation as better than it is, believing that they could still drive. That is, respondents may overestimate their driving ability more than their actual rating or underestimate their driving avoidance frequency. Therefore, detailed diagnostics by professionals are required for individuals identified as must-watch drivers, particularly those like must-watch drivers A-C in Figure 4 who reported better health conditions than the same age group. Driving cessation is not determined simply by health conditions, so it is necessary to assess each individual current driver. The individual diagnosis of must-watch drivers and preliminary group by professionals would be required for age-related factors that cannot be obtained as information in the Needs Survey.

Although the questionnaire was sent to all older adult residents in Tsurui Village and achieved a sufficient response rate of 47.4% for a comprehensive understanding of older adult individuals, respondents may not be collected from not only healthy older adults who were not interested in answering but also older adults with health conditions that make it difficult to answer the questions. Although this survey was conducted in Tsurui Village, a rural area, the projection of driving avoidance by the KCL would require confirmation by the same questionnaire in the city or its suburbs due to the unique regional characteristics of Tsurui Village.

## 5. Conclusions

In rural areas with limited public transportation, it is crucial for older adults to drive to maintain their daily lives. Therefore, assessing whether older adults are forced to give up driving is essential to provide them with appropriate administrative services.

The “Public Survey of Long-Term Care Prevention and Needs in Spheres of Daily Life (Needs Survey)” is conducted triennially by local governments across Japan, including the “*Kihon* checklist (KCL)” widely used in the welfare field. This study demonstrated the possibility of grasping the older adults’ driving status based on the Needs Survey (particularly the KCL) and conducted a questionnaire survey in Tsurui Village, a rural area in Japan, which combined KCL with self-perceptions about driving utilized in previous research.

Based on the responses, a logistic regression model was constructed using sociodemographic factors and health conditions indicated by KCL to guess the likelihood of not driving for both current and former drivers. The results revealed that the likelihood of not driving depends significantly on health conditions under the same age and gender. Although many respondents within the same age and gender groups scored below 0.5, some individuals scored above 0.5 and were classified as must-watch drivers [31]. In other words, the current drivers with relatively poor health among groups with a low likelihood of not driving, as well as those with relatively good health among groups with a high likelihood of not driving, are identified as must-watch drivers. This study demonstrated that the likelihood of not driving provides more detailed information on the driving status of individual older drivers, rather than the 0.5 must-watch drivers used in He et al. [31]. The likelihood can be used to identify older drivers who are slightly below the threshold of 0.5, the preliminary group that will become must-watch drivers if the conditions change slightly, and to judge the severity of “must-watch drivers” by the degree to which it exceeds the threshold of 0.5.

Furthermore, while previous studies used the self-rating of driving ability to analyze daily driving challenge situations, such as night driving [15], this study also demonstrated that KCL could be used instead of the self-rating of driving ability, and consistent results would be obtained in multiple regression analysis.

KCL proved to be a valuable tool for assessing the driving conditions of older drivers. In Japan, it is possible to regularly assess the driving conditions of older adults nationwide by utilizing the Needs Survey, which allows us to assess the current conditions of older adults, such as their daily lifestyle and economic situation, as well as their age, place of residence, and family structure. As an implication of this study, administrations would be recommended to initially use KCL as a screening tool for the entire population of older drivers. As KCL is based on the self-perceptions of older adults as described in the last paragraph as a limitation above, they would identify a small number of individuals required for close monitoring.

Subsequently, professionals should evaluate whether these individuals should be required to stop driving. Additionally, the support for older adults who have ceased driving should not be based on the entire population’s opinions but instead tailored to the needs of the relatively small number of older adults in urgent situations.

## Figures and Tables

**Table 1:** Demographic data on current drivers, former drivers, and non-drivers (*n* = 393).

Respondents (*n* = 393)	Current Drivers (*n* = 317)		Former Drivers (*n* = 33)		Non-Drivers (*n* = 43)	
Gender						
Male	169	53.3%	12	36.4%	3	7.0%
Female	148	46.7%	21	63.6%	40	93.0%
Age						
65–69	96	30.3%	0	0.0%	2	4.7%
70–74	102	32.2%	3	9.1%	6	14.0%
75–79	59	18.6%	4	12.1%	7	16.3%
80–84	40	12.6%	13	39.4%	6	14.0%
85–89	15	4.7%	3	9.1%	11	25.6%
90–94	3	0.9%	6	18.2%	6	14.0%
95–99	2	0.6%	4	12.1%	5	11.6%
Region (bus)						
Yes	231	72.9%	25	75.8%	33	76.7%
No	86	27.1%	8	24.2%	10	23.3%
Living alone						
Yes	62	19.6%	15	45.5%	14	32.6%
No	255	80.4%	18	54.5%	29	67.4%
Frailty Measures						
Robust	104	32.8%	5	15.2%	7	16.3%
Pre-frailty	139	43.8%	10	30.2%	9	20.9%
Frailty	74	23.3%	18	54.5%	27	62.8%
KCL scores						
Mean (SD)	5.44 (3.60)		9.58 (6.12)		9.67 (5.87)	
Median [Min, Max]	5.00 [0, 21.0]		9.00 [0, 23.0]		8.00 [0, 23.0]	

**Table 2:** Predicting whether to drive or not using logistic regression analysis.

	Model 1	Model 2	Model 3
Age (5-year) OR (95%CI)	3.056 *** (2.612–6.776)	3.187 *** (2.255–4.834)	3.263 *** (2.338–4.860)
Gender			
Male OR (95%CI)	1.000 (Ref)	1.000 (Ref)	1.000 (Ref)
Female OR (95%CI)	6.011 ** (1.989–21.763)	5.452 ** (1.848–18.855)	4.719 ** (1.712–14.723)
Living alone			
Yes OR (95%CI)	2.616 (0.985–7.029)	2.687 * (1.018–7.199)	2.659 * (1.052–6.784)
No OR (95%CI)	1.000 (Ref)	1.000 (Ref)	1.000 (Ref)
KCL scores OR (95%CI)	1.160 ** (1.059–1.277)		
Frailty Measures			
Robust OR (95%CI)		1.000 (Ref)	
Pre-frailty OR (95%CI)		1.108 (0.327–4.104)	
Frailty OR (95%CI)		3.875 * (1.215–14.126)	
AIC	139.610	144.200	147.830

Note: * *p* < 0.05; ** *p* < 0.01; *** *p* < 0.001; OR: Odds Ratio; CI: Confidence Interval; KCL scores: *Kihon* Checklist; AIC: Akaike information criterion; Statistical powers (1-β) in Models 1 to 3 are over 0.99 using G*Power3.1 [47].

**Table 3:** Driving status by logistic analysis of Models 1, 2, 3 for current/former drivers.

	Model 1		Model 2		Model 3		
	Driving	Not driving	Driving	Not driving	Driving	Not driving	Total
Current	311	6	311	6	314	3	317
Former	21	12	22	11	25	8	33
Total	332	18	333	17	339	11	350

**Table 4:** Riding in a car driven by others, self-rating of driving ability, the importance of driving for daily life, and current driving frequency by gender for current drivers.

	Male	Female	*p*-Value
	n = 169	n = 148	
Riding in a car driven by others			<0.001 ^a^
No	158 (93.5%)	113 (76.4%)	
Yes	11 (6.5%)	35 (23.6%)	
Self-rating of driving ability			0.066 ^a^
Excellent	14 (8.3%)	6 (4.1%)	
Good	23 (13.6%)	16 (10.8%)	
Average	112 (66.3%)	92 (62.2%)	
Fair	17 (10.1%)	30 (20.3%)	
Poor	3 (1.8%)	4 (2.7%)	
Evaluate the importance of driving			0.011 ^b^
Very important	121 (71.6%)	82 (55.4%)	
Important	38 (22.5%)	51 (34.5%)	
Somewhat important	10 (5.9%)	11 (7.4%)	
Not very important	0 (0%)	3 (2.0%)	
Not important at all	0 (0%)	1 (0.7%)	
Current driving frequency			<0.001 ^a^
Nearly daily	86 (50.9%)	23 (15.5%)	
3–5 times weekly	44 (26.0%)	63 (42.6%)	
1–2 times weekly	32 (18.9%)	45 (30.4%)	
2–3 times monthly	6 (3.6%)	17 (11.5%)	
Not driven in the past six months	1 (0.6%)	0 (0%)	

Note: a, Chi-Squared Test; b, Fisher’s Exact Test.

**Table 5:** Spearman’s rank correlation of driving avoidance frequency between seven challenging driving situations for each respondent.

	Challenging Daily-Life Driving Situations				Challenging Non-Daily-Life Driving Situations		
	Rain	Nights	Dawn or Dusk	Nights During Rain	Long Time	First Time in Place	Highways
rain	1	0.71	0.63	0.70	0.54	0.58	0.49
nights		1	0.79	0.89	0.60	0.64	0.56
dawn or dusk			1	0.77	0.62	0.64	0.55
nights during rain				1	0.63	0.64	0.57
long time					1	0.77	0.66
first time in place						1	0.72
highways							1

**Table 6:** Partial regression coefficients and confidence intervals for the explanatory variables in multiple regression analyses using Models A, B, C, and D.

	Model A	Model B	Model C	Model D
Female	2.926 ***	2.822 ***	3.195 ***	3.155 ***
(95% CI)	(2.139, 3.712)	(2.017, 3.627)	(2.396, 3.995)	(2.318, 3.991)
Age	0.425 **	0.456 **	0.389 *	0.419 *
(95% CI)	(0.119, 0.731)	(0.143, 0.770)	(0.074, 0.703)	(0.090, 0.748)
Living alone, yes	1.100 *	1.104 *	1.113 *	1.125 *
(95% CI)	(0.129, 2.071)	(0.108, 2.100)	(0.114, 2.113)	(0.080, 2.170)
Self-rating of driving ability	1.161 ***	1.487 ***		
(95% CI)	(0.642, 1.681)	(0.979, 1.996)		
KCL scores	0.236 ***		0.310 ***	
(95% CI)	(0.124, 0.348)		(0.200, 0.420)	
R^2^	0.291	0.251	0.247	0.173
Adjusted R^2^	0.279	0.242	0.237	0.165
AIC	1697.004	1712.060	1713.992	1741.777

Note: * *p* < 0.05; ** *p* < 0.01; *** *p* < 0.001; AIC: Akaike information criterion; CI: Confidence Interval; Statistical powers (1-β) in Models A to D are over 0.99 using G*Power3.1 [47].

**Table 7:** The conditions related to driving for must-watch, former, and current (except must-watch drivers) drivers and their imagined future conditions.

	BusRoute Area	Live Alone	KCL Scores	SRDA	EID	TS DAF	Usual Driving Destination		Current Using Transportation		Future Using Transportation *	
							Kushiro	Village	Others	Public *	Others	Public *
Must-watch drivers (n = 6)	17%	33%	9.6	3.5	1.5	11	83%	83%	33%	0%	67%	67%
Former drivers (n = 33)	24%	45%	10.8						76%	58%	79%	42%
Other current drivers (n = 311)	27%	19%	5.3	2.9	1.5	9	86%	64%	14%	4%	56%	83%
*p-value*	0.945 ^a^	0.001 ^a^	<0.001 ^b^	0.118 ^c^	0.981 ^c^	0.322 ^c^	1.000 ^a^	0.427 ^a^	<0.001 ^a^	<0.001 ^a^	0.028 ^a^	<0.001 ^a^

Note: a, Fisher’s Exact Test; b, Kruskal-Walli’s test; c, Wilcoxon rank sum test; SRDA is self-rating of driving ability; EID is evaluating the importance of driving; TSDAF is the “total score of daily-life driving avoidance frequency”; “Future using Transportation *” is their current-imagined transportations after their driving cessation in the future. Public * is public transportation, including buses, taxis, shuttles to and from the clinic, and volunteer-driving cars. Percentages mean the ratio of respondents choosing those items when total must-watch, former, and current (except must-watch drivers) drivers are 100%: living on a bus route area, driving to a destination in Kushiro city or village, riding in a car driven by others, or using a bus. As former drivers do not drive, there is no data on items related to driving.

## Data Availability

These data are inappropriate for public disclosure due to ethical issues. If you are a researcher interested in an analysis using these data, please request access to the confidential data from the bioethics review committee at the Graduate School of Env. Science of Env. Earth Science in Hokkaido University.

## Appendix A

The “*Kihon* Checklist (KCL)” was introduced by the Japanese Ministry of Health, Labor, and Welfare in 2006 to identify vulnerable older adults at a higher risk of becoming dependent. The English version of the KCL was translated from the original Japanese into Table A1 [42,43].

In Table A2, Model E was used with all information as explanatory variables in the multiple regression analysis: respondents’ gender, age, bus route area, living alone status, KCL, riding in a car driven by others, self-rating of driving ability, evaluation of the importance of driving for their living, and current driving frequency. Model A was adopted as statistically significant from all in Model E. The correlation coefficient of results in Model E is higher than those in Model A due to using all information. However, as for the adjusted correlation coefficient and AIC values, those in Model A are better than those in Model E. Model E has similar results, slightly not better than those in Model A.

**Table A1:** The *Kihon* checklist [42,43].

No.	Questions	Answer	
1	Do you go out by bus or train by yourself?	0. YES	1. NO
2	Do you go shopping to buy daily necessities by yourself?	0. YES	1. NO
3	Do you manage your own deposits and savings at the bank?	0. YES	1. NO
4	Do you sometimes visit your friends?	0. YES	1. NO
5	Do you turn to your family or friends for advice?	0. YES	1. NO
6	Do you normally climb stairs without using handrail or wall for support?	0. YES	1. NO
7	Do you normally stand up from a chair without any aids?	0. YES	1. NO
8	Do you normally walk continuously for 15 min?	0. YES	1. NO
9	Have you experienced a fall in the past year?	1. YES	0. NO
10	Do you have a fear of falling while walking?	1. YES	0. NO
11	Have you lost 2 kg or more in the past 6 months?	1. YES	0. NO
12	Height: cm, weight: kg, BMI: kg/m2 If BMI is less than 18.5, this item is scored.	1. YES	0. NO
13	Do you have any difficulties eating tough foods compared to 6 months ago?	1. YES	0. NO
14	Have you choked on your tea or soup recently?	1. YES	0. NO
15	Do you often experience having a dry mouth?	1. YES	0. NO
16	Do you go out at least once a week?	0. YES	1. NO
17	Do you go out less frequently compared to last year?	1. YES	0. NO
18	Do your family or your friends point out your memory loss? e.g., “You ask the same question over and over again.”	1. YES	0. NO
19	Do you make a call by looking up phone numbers?	0. YES	1. NO
20	Do you find yourself not knowing today’s date?	1. YES	0. NO
21	In the last 2 weeks have you felt a lack of fulfilment in your daily life?	1. YES	0. NO
22	In the last 2 weeks have you felt a lack of joy when doing the things you used to enjoy?	1. YES	0. NO
23	In the last 2 weeks have you felt difficulty in doing what you could do easily before?	1. YES	0. NO
24	In the last 2 weeks have you felt helpless?	1. YES	0. NO
25	In the last 2 weeks have you felt tired without a reason?	1. YES	0. NO

**Table A2:** Results from all of the multiple regression analyses.

	Model A	Model B	Model C	Model D	Model E
Female	2.926 ***	2.822 ***	3.195 ***	3.155 ***	2.688 ***
	(2.139, 3.712)	(2.017, 3.627)	(2.396, 3.995)	(2.318, 3.991)	(1.835, 3.541)
Age	0.425 **	0.456 **	0.389 *	0.419 ***	0.416 **
	(0.119, 0.731)	(0.143, 0.770)	(0.074, 0.703)	(0.090, 0.748)	(0.107, 0.725)
Living alone, yes	1.100 *	1.104 *	1.113 *	1.125 *	1.220 *
	(0.129, 2.071)	(0.108, 2.100)	(0.114, 2.113)	(0.080, 2.170)	(0.231, 2.209)
Self-rating of driving ability	1.161 ***	1.487 ***			1.090 ***
	(0.642, 1.681)	(0.979, 1.996)			(0.561, 1.619)
KCL scores	0.236 ***		0.310 ***		0.217 ***
	(0.124, 0.348)		(0.200, 0.420)		(0.104, 0.331)
Bus route area					0.685
					(−0.185, 1.556)
Riding in a car driven by others					0.046
					(−1.133, 1.226)
Current driving frequency					0.266
					(−0.192, 0.723)
Evaluate the importance of driving					0.228
					(−0.389, 0.846)
R^2^	0.291	0.251	0.247	0.173	0.302
Adjusted R^2^	0.279	0.242	0.237	0.165	0.281
AIC	1697.004	1712.06	1713.992	1741.777	1699.912

Note: * *p* < 0.05; ** *p* < 0.01; *** *p* < 0.001; Models A, B, C, and D are the same in Table 6.

## References

[1] United Nations Department of Economic and Social Affairs, Population Division 2022 World Population Prospects 2022: Summary of Results. 2022. UN DESA/POP/2022/TR/NO. 3. https://www.un.org/development/desa/pd/sites/www.un.org.development.desa.pd/files/wpp2022_summary_of_results.pdf.

[2] The 9th International Comparative Survey on the Lives and Attitudes of the Older Adults. https://www8.cao.go.jp/kourei/ishiki/r02/zentai/pdf_index.html.

[3] Jeong S.H., Kim E.Y., Lee S.J., Choi W.J., Oh C., Sung H.J., Kim J. (2023). Health Status and Activity Discomfort among Elderly Drivers: Reality of Health Awareness. Healthcare.

[4] Green K.A., McGwin G., Owsley C. (2013). Associations Between Visual, Hearing, and Dual Sensory Impairments and History of Motor Vehicle Collision Involvement of Older Drivers. J. Am. Geriatr. Soc..

[5] Hill L.L., Andrews H., Li G.H., DiGuiseppi C.G., Betz M.E., Strogatz D., Pepa P., Eby D.W., Merle D., Kelley-Baker T. (2020). Medication use and driving patterns in older drivers: Preliminary findings from the LongROAD study. Inj. Epidemiol..

[6] Young K.L., Stephens A.N., McDonald H. (2024). Executive function and drivers’ ability to self-regulate behaviour when engaging with devices. Curr. Psychol..

[7] Tinella L., Lopez A., Caffò A.O., Nardulli F., Grattagliano I., Bosco A. (2021). Cognitive Efficiency and Fitness-to-Drive along the Lifespan: The Mediation Effect of Visuospatial Transformations. Brain Sci..

[8] Svetina M. (2016). The reaction times of drivers aged 20 to 80 during a divided attention driving. Traffic Inj. Prev..

[9] Kim Y.S., Shin H., Um S. (2023). The Subjective Experiences of Driving Cessation and Life Satisfaction. Behav. Sci..

[10] Ishii H., Doi T., Tsutsumimoto K., Nakakubo S., Kurita S., Shimada H. (2021). Driving cessation and physical frailty in community-dwelling older adults: A longitudinal study. Geriatr. Gerontol. Int..

[11] Edwards J.D., Lunsman M., Perkins M., Rebok G.W., Roth D.L. (2009). Driving Cessation and Health Trajectories in Older Adults. J. Gerontol. Ser. A Biol. Sci. Med. Sci..

[12] Liang D., Lau N., Antin J.F. (2022). Modeling of older adults? driving exposure and avoidance using objective driving data in a naturalistic driving study. Accid. Anal. Prev..

[13] Vivoda J.M., Molnar L.J., Eby D.W., DiGuiseppi C., Jones V., Li G.H., Strogatz D., Yung R.Y., Nyquist L., Smith J. (2022). All are not created equal: Assessing initial driving self-regulation behaviors among older adults. J. Transp. Health.

[14] Moták L., Gabaude C., Bougeant J.C., Huet N. (2014). Comparison of driving avoidance and self-regulatory patterns in younger and older drivers. Transp. Res. Part F Traffic Psychol. Behav..

[15] Charlton J.L., Oxley J., Fildes B., Oxley P., Newstead S., Koppel S., O’Hare M. (2006). Characteristics of older drivers who adopt self-regulatory driving behaviours. Transp. Res. Part F Traffic Psychol. Behav..

[16] Bergen G., West B.A., Luo F., Bird D.C., Freund K., Fortinsky R.H., Staplin L. (2017). How do older adult drivers self-regulate? Characteristics of self-regulation classes defined by latent class analysis. J. Saf. Res..

[17] Molnar L.J., Eby D.W., Charlton J.L., Langford J., Koppel S., Marshall S., Man-Son-Hing M. (2013). Driving avoidance by older adults: Is it always self-regulation?. Accid. Anal. Prev..

[18] Ross L.A., Clay O.J., Edwards J.D., Ball K.K., Wadley V.G., Vance D.E., Cissell G.M., Roenker D.L., Joyce J.J. (2009). Do Older Drivers At-Risk for Crashes Modify Their Driving Over Time?. J. Gerontol. Ser. B-Psychol. Sci. Soc. Sci..

[19] Zhu Y.F., Jiang M.L., Yamamoto T. (2024). Does a cautious driving style reduce the crash risk of older drivers? An analysis using a novel driving style recognition method. Transp. Res. Part F-Traffic Psychol. Behav..

[20] Feng Y.R., Meuleners L. (2020). Planning for driving cessation in older drivers. Transp. Res. Part F-Traffic Psychol. Behav..

[21] Zeitler E., Buys L. (2015). Mobility and out-of-home activities of older people living in suburban environments: ‘Because I’m a driver, I don’t have a problem’. Ageing Soc..

[22] Tinella L., Bosco A., Traficante S., Napoletano R., Ricciardi E., Spano G., Lopez A., Sanesi G., Bergantino A.S., Caffò A.O. (2023). Fostering an Age-Friendly Sustainable Transport System: A Psychological Perspective. Sustainability.

[23] Current Population Estimates as of 1 October 2022, Statistics Bureau of Japan. https://www.stat.go.jp/english/data/jinsui/2022np/index.html.

[24] National Police Agency (2023). Driver’s License Statistics. https://www.npa.go.jp/publications/statistics/koutsuu/menkyo.html.

[25] Abe T., Seol J., Kim M., Okura T. (2018). The relationship of car driving and bicycle riding on physical activity and social participation in Japanese rural areas. J. Transp. Health.

[26] Uchida K., Ueda Y., Nakamura J., Murata S., Endo T., Otani K., Ono R. (2023). Effect of car use on social frailty among community-dwelling older adults in rural areas. J. Transp. Health.

[27] Abe T., Kitamura A., Seino S., Yokoyama Y., Amano H., Taniguchi Y., Nishi M., Nofuji Y., Ikeuchi T., Sugiyama T. (2020). Frailty Status and Transport Disadvantage: Comparison of Older Adults’ Travel Behaviours between Metropolitan, Suburban, and Rural Areas of Japan. Int. J. Environ. Res. Public Health.

[28] Yamada M., Arai H. (2020). Long-Term Care System in Japan. Ann. Geriatr. Med. Res..

[29] Ministry of Health, Labor and Welfare (2009). Manual on Assessment of Lifestyle Function for Care Prevention (Revised Edition). http://www.mhlw.go.jp/topics/2009/05/dl/tp0501-1c.pdf.

[30] Ministry of Health, Labor and Welfare The Public Survey of Long-Term Care Prevention and Needs in Spheres of Daily Life. https://www.mhlw.go.jp/content/12301000/000532246.pdf.

[31] He M., Takamatu K., Kishi K., Yamanaka Y. (2020). An empirical analysis of the driving status for elderly people using the survey for “Insured Long-Term Care Service Plans”. J. City Plan. Inst. Jpn..

[32] Liu J., Fujii Y., Fujii K., Seol J., Kim M., Tateoka K., Nagata K., Zhang H.L., Okura T. (2022). Pre-frailty associated with traffic crashes in Japanese community-dwelling older drivers. Traffic Inj. Prev..

[33] Baldock M.R.J., Mathias J.L., McLean A.J., Berndt A. (2006). Self-regulation of driving and its relationship to driving ability among older adults. Accid. Anal. Prev..

[34] St Louis R.M., Koppel S., Molnar L.J., Di Stefano M., Darzins P., Bedard M., Mullen N., Myers A., Marshall S., Charlton J.L. (2020). The relationship between psychological resilience and older adults’ self-reported driving comfort, abilities, and restrictions. J. Transp. Health.

[35] Sümer N. (2003). Personality and behavioral predictors of traffic accidents:: Testing a contextual mediated model. Accid. Anal. Prev..

[36] Tinella L., Bosco A., Koppel S., Lopez A., Spano G., Ricciardi E., Traficante S., Napoletano R., Grattagliano I., Caffo A.O. (2024). Sociodemographic and psychological factors affecting motor vehicle crashes (MVCs): A classification analysis based on the contextual-mediated model of traffic-accident involvement. Curr. Psychol..

[37] D’Ambrosio L.A., Donorfio L.K.M., Coughlin J.F., Mohyde M., Meyer J. (2008). Gender differences in self-regulation patterns and attitudes toward driving among older adults. J. Women Aging.

[38] Choi M., Adams K.B., Kahana E. (2013). Self-Regulatory Driving Behaviors: Gender and Transportation Support Effects. J. Women Aging.

[39] Blanchard R.A., Myers A.M. (2010). Examination of driving comfort and self-regulatory practices in older adults using in-vehicle devices to assess natural driving patterns. Accid. Anal. Prev..

[40] Strogatz D., Mielenz T.J., Johnson A.K., Baker I.R., Robinson M., Mebust S.P., Andrews H.F., Betz M.E., Eby D.W., Johnson R.M. (2020). Importance of Driving and Potential Impact of Driving Cessation for Rural and Urban Older Adults. J. Rural. Health.

[41] Ackerman M.L., Crowe M., Vance D.E., Wadley V.G., Owsley C., Ball K.K. (2011). The Impact of Feedback on Self-rated Driving Ability and Driving Self-regulation Among Older Adults. Gerontologist.

[42] Arai H., Satake S. (2015). English translation of the Kihon Checklist. Geriatr. Gerontol. Int..

[43] Fukutomi E., Okumiya K., Wada T., Sakamoto R., Ishimoto Y., Kimura Y., Kasahara Y., Chen W.L., Imai H., Fujisawa M. (2013). Importance of cognitive assessment as part of the “Kihon Checklist” developed by the Japanese Ministry of Health, Labor and Welfare for prediction of frailty at a 2-year follow up. Geriatr. Gerontol. Int..

[44] Satake S., Senda K., Hong Y.J., Miura H., Endo H., Sakurai T., Kondo I., Toba K. (2016). Validity of the Kihon Checklist for assessing frailty status. Geriatr. Gerontol. Int..

[45] Fried L.P., Tangen C.M., Walston J., Newman A.B., Hirsch C., Gottdiener J., Seeman T., Tracy R., Kop W.J., Burke G. (2001). Frailty in older adults: Evidence for a phenotype. J. Gerontol. Ser. A-Biol. Sci. Med. Sci..

[46] R Core Team (2022). R: A Language and Environment for Statistical Computing.

[47] Faul F., Erdfelder E., Buchner A., Lang A.-G. (2009). Statistical power analyses using G*Power 3.1: Tests for correlation and regression analyses. Behav. Res. Methods.

